# Rivaroxaban Modulates TLR4/Myd88/NF-Kβ Signaling Pathway in a Dose-Dependent Manner With Suppression of Oxidative Stress and Inflammation in an Experimental Model of Depression

**DOI:** 10.3389/fphar.2021.715354

**Published:** 2021-09-24

**Authors:** Walaa Yehia Abdelzaher, Hanaa H. Mohammed, Nermeen N. Welson, Gaber El-Saber Batiha, Roua S. Baty, Asmaa Mohamed Abdel-Aziz

**Affiliations:** ^1^ Department of Pharmacology, Faculty of Medicine, Minia University, Minia, Egypt; ^2^ Department of Histology, Faculty of Medicine, Minia University, Minia, Egypt; ^3^ Department of Forensic Medicine and Clinical Toxicology, Faculty of Medicine, Beni-Suef University, Beni-Suef, Egypt; ^4^ Department of Pharmacology and Therapeutics, Faculty of Veterinary Medicine, Damanhour University, Damanhour, Egypt; ^5^ Department of Biotechnology, College of Science, Taif University, Taif, Saudi Arabia

**Keywords:** depression, MyD88, NF-kβ, rivaroxban, TLR4

## Abstract

Depression is a common mental illness leading to upset or anxiety, with a high incidence rate in the world. Depression can lead to suicidal thoughts and behavior. The present study aimed to evaluate the effect of the direct oral anticoagulant rivaroxaban (RVX), in the model of depression induced by chronic unpredicted mild stress (CUMS) in rats. Fifty-six male Wister rats were randomly divided into seven experimental groups (8 rats/group); Group 1: Control group given vehicle per oral (p.o.), Group 2: RVXL-control group (received rivaroxaban 20 mg/kg/day, p.o..), Group 3: RVXH-control group (received rivaroxaban 30 mg/kg/day, p.o.), Group 4: chronic unpredictable mild stress (CUMS) group, Group 5: FLX-treated CUMS group (received fluoxetine 10 mg/kg/day, p.o..), Group 6: RVXL-treated CUMS group (received rivaroxaban 20 mg/kg/day, p.o.), and Group 7: RVXH-treated CUMS group (received rivaroxaban 30 mg/kg/day, p.o.). The rats received the drugs from the first day of the experiment and continued till 4 weeks—the duration of the study. The following were measured: monoamine neurotransmitters, malondialdehyde (MDA), total nitrite/nitrate (NOx), reduced glutathione (GSH), superoxide dismutase (SOD), Toll-like receptor 4 (TLR4), myeloid differentiation factor 88 (MyD88), nuclear factor‐kappa B (NF‐κB), tumor necrosis factor-α (TNF-α), brain-derived neurotrophic factor (BDNF), and vascular endothelial growth factor-A (VEGF-A). A forced swimming test (FST) was done. Furthermore, histological changes and glial fibrillary acidic protein (GFAP) immunoexpression were evaluated. CUMS showed a significant decrease in hypothalamic neurotransmitters, hippocampal GSH, SOD, BNDF, and VEGF-A with a significant increase in hippocampal MDA, NOx, NF-kβ, Myd88, TLR4, TNF-α, and GFAP immunoexpression. RVX showed significant improvement in all parameters (*p* **-**value < 0.0001). In conclusion, RVX in a dose-dependent manner possesses potent ameliorative effects against depression by reducing the oxidative stress and inflammatory process, through the regulation of the TLR4/Myd88/NF-kβ signaling pathway.

## Introduction

Depression is considered a common mental illness leading to upset or anxiety, with a high incidence rate in the world. Depression may lead to suicidal thoughts and behavior, or mental illness manifestations, for example, fantasy or delusion (Sun et al., 2020). The World Health Organization statistics show that more than three hundred million people around the world (4.4%) endure from depression (World Health Organization (WHO)., 2017).

The pathophysiological mechanisms of depression are not well identified till date, but many studies suggested that the impairment of intracerebral neurotransmission, mainly serotonin (5-HT), norepinephrine (NE), and dopamine (DA) is mainly responsible for the evolution of depression (Du et al., 2020).

Moreover, inflammation also plays an axial role in subserving depression symptom phenomenology. Patients who received interferon-α or interleukin (IL)-2 therapies have developed depressive symptoms. Otherwise, some cytokines, for example, tumor necrosis factor-α (TNF-α) that is related to major depressive disorder, are implicated in the regulation of brain NE or 5-HT (Warner-Schmidta et al., 2011; Lu et al., 2017).

Many animal studies showed that the exposure to external or internal stress leads to the activation of innate inflammatory immune reaction and the dysfunction of immune cells’ production of inflammatory cytokines causing sickness behavior and anhedonia (Slavich and Irwin, 2014; Lu et al., 2017).

Toll-like receptors (TLRs) are recognition proteins of the innate immunity which are found in the circumventricular organs and the choroid plexus (Dantzer et al., 2008). Stimulation of TLR4 leads to the recruitment of inflammatory cells *via* myeloid differentiation factor 88 (MyD88) that ultimately stimulates the release of the proinflammatory mediators like nuclear factor-kappa B (NF-κB), TNF-α, and IL-1β (Yang et al., 2019; Zhong et al., 2019).

Brain-derived neurotrophic factor (BDNF) is the most prominent neurotrophic factor which shows decreased expression and function with depression and reveals an improvement by the antidepressant therapy (Zhou et al., 2017; Sun et al., 2020). The BDNF protein along with mRNA in the hippocampus of mice with depression was significantly reduced by learned helplessness (Su et al., 2016).

It has been well established that the inflammatory and coagulation pathways are invariably linked. Various coagulation proteases trigger a diversity of proinflammatory responses, in addition to their critical role in the coagulation cascade, such as factor Xa (Zuo et al., 2015).

The factor Xa is a clef coagulation factor in thrombin generation and clot formation which induces intracellular signaling via the proteolytic cleavage of proteinase-activated receptors. Furthermore, it enhances the expression of IL-6 and IL-8 and stimulates the proliferation of fibroblasts and smooth muscle cells (Utku et al., 2015).

Overall, it becomes increasingly clear that the deposition of different coagulation factors in the CNS tissue may trigger the exacerbation of inflammation, thereby limiting regenerative mechanisms. Interestingly, the binding of coagulation factors to their cellular targets is usually independent of their protease function. So, the targeted inhibition of the coagulation factors that facilitate disease pathogenesis without affecting their protease activity represents an ideal strategy for pharmacological intervention in different neuroinflammatory disorders without unwarranted side effects like bleeding (Göbel et al., 2018).

Rivaroxaban (RVX) is well known for selectively and competitively blocking thrombin generation *via* preventing the transformation of prothrombin to thrombin. It is a direct oral inhibitor of the factor Xa. It has a similar efficacy to the standard therapy and is associated with a significantly reduced risk of bleeding (Robertson and Kesteven., 2014). RVX has antioxidant (Al-Harbi et al., 2020) and anti-inflammatory (Jhaveri et al., 2011) effects. RVX could protect the experimental animals from inflammation by blocking factor Xa, which is considered as an accelerator for the generation of the proinflammatory mediators (Jhaveri et al., 2011; Al-Harbi et al., 2020). Also, Dittmeier and his coworkers (2016) found that the pretreatment with RVX attenuates stroke severity in rats by a dual antithrombotic and anti-inflammatory mechanism.

This experiment aimed to estimate the effect of the direct oral anticoagulant RVX which has antioxidant and anti-inflammatory properties in the model of depression induced by chronic unpredicted mild stress (CUMS) in rats. RVX may reduce NF-κB activity through the Myd88-dependent pathway of TLR4, hence it is assumed to produce an antidepressant-like response reducing the expression and release of downstream proinflammatory cytokines.

## Material and Methods

### Drugs and Chemicals

RVX was purchased from Inspire Pharmaceutical Company (Cairo, Egypt) and fluoxetine (FLX) was purchased from Amoun Pharmaceutical Industries Company (Cairo, Egypt). Both drugs were dissolved in a saline vehicle (Hodges et al., 2013; Liu et al., 2018). All other chemicals were of analytical grade and were purchased from commercial sources.

### Animals

Adult male Wistar rats weighing 200–240 g, aged 8–10 weeks, were acquired from the National Research Center, Cairo, Egypt. The rodents were left for acclimatization in their cages (4 rats/cage) in a normal light–dark cycle, with free access to tap water and normal diet (El-Nile Company, Egypt) for 2 weeks before initiating the experiment. All stressors were applied to animals outside their housing area in a separate room. The study procedures complied with the ARRIVE guidelines, the U.K. Animals Act (1986), the EU Directive, and the Ethical Board of Faculty of Medicine, Minia University, which approved the procedures of the animal experiment protocol (277:7/2019).

### Experimental Design

**FIGURE 1:**
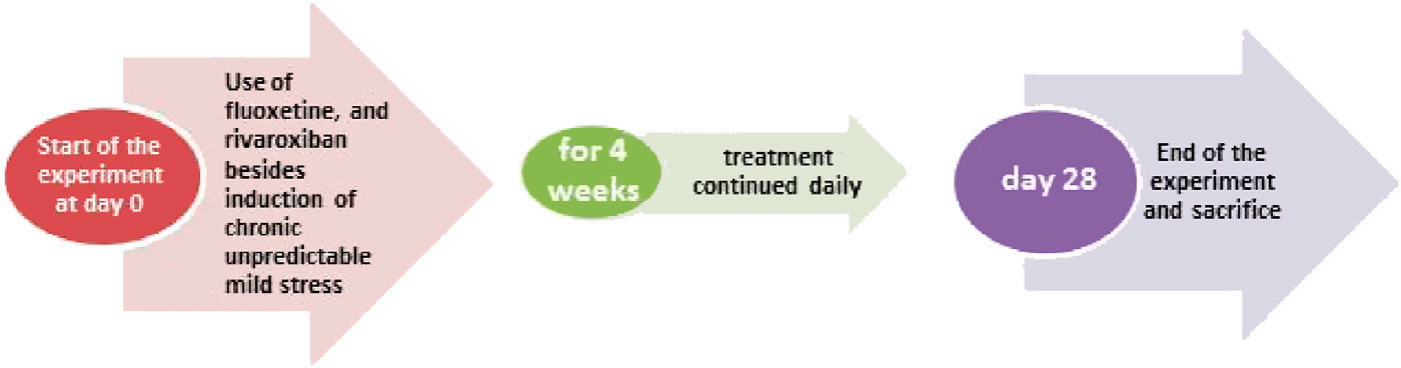
Diagrammatic scheme of the experimental design.

Fifty-six male Wistar rats were assigned into seven experimental groups (8 rat/group): Group 1: Control group given vehicle (p.o.); Group 2: RVXL-control group (received rivaroxaban 20 mg/kg/day, p.o.) (Vilaseca et al., 2017),; Group 3: RVXH-control group (received rivaroxaban 30 mg/kg/day, p.o.) (Liu et al., 2018); Group 4: chronic unpredictable mild stress (CUMS) group; Group 5: FLX-treated CUMS group (received fluoxetine 10 mg/kg/day, p. o.) (Szewczyk et al., 2019); Group 6: RVXL-treated CUMS group (received rivaroxaban 20 mg/kg/day, p. o.); and Group 7: RVXH-treated CUMS group (received rivaroxaban 30 mg/kg/day, p. o.), the rats received the drugs from the first day of the experiment and it continued till 4 weeks; the duration of the study (Chen et al., 2019) (Figure 1). At the end of the experiment, the rodents were killed, the brain tissues were excised, and the hippocampal parts were instantly stored at −80°C for biochemical assay.

### Chronic Unpredictable Mild Stress Procedure

Chronic exposure of the rats to differently scheduled stressors was done. The stressors were administered once daily (at the same time) for 4 weeks with 7 days in between before repetitions (presented in a random order from week to week). Every stressor was applied 4 times within 28 days. The stressors were applied to prompt a depressive state, involving 1) social crowding (8 rats/cage, 24 h); 2) swimming at 30°C for 20 min; 3) in a cage tilted at 30° from the horizontal (24 h); 4) swimming at 8°C–10°C for 5 min; 5) in a wet cage for 24 h; 6) tail pinch for 2 min; and 7) food and water deprivation for 24 h (Yang et al., 2017).

### Sample Collection and Storage

Animals’ decapitation after IP injection of urethane hydrochloride (1 g/kg i.p.) anesthesia was performed (Abdel-Gaber et al., 2015). Careful excision of the brain tissue was done. The hippocampal tissue areas were fixed in 10% formalin and processed for paraffin sections for histopathological and immunohistochemical assessments, and other specimens were homogenized in 20% w/v cold potassium phosphate buffer (0.01 M, pH 7.4), centrifuged at 5,000 rpm for 10 min at 4°C, and the resulting supernatant was kept for the assessment of other biochemical parameters.

### Neurotransmitter Analysis

Concentrations of NE, DA, and 5-HT were determined in the hippocampal tissue by the spectrophotofluorometric method (Shimadzu RF-5000). Transmitter’s oxidations were done by adding 0.1 N iodine followed by stopping the oxidation by alkaline sulfite addition to produce specific fluorescence. The induced fluorescence was quantified at a particular emission wavelength following the excitation at another specific wavelength that varies according to the transmitter. The intensity of the fluorescence is directly proportional to the neurotransmitter level in the brain tissue (Aziz et al., 2013).

### Measurement of Hippocampal Oxidative Stress Parameters

SOD activity was colorimetrically quantified at 420 nm, and the reduced glutathione (GSH) assessment relied on the fact that the sulfhydryl groups of GSH react with 5,5-dithiol-bis-2-nitrobenzoic acid (Ellman’s reagent) giving a yellow-colored 5-thio-2-nitrobenzoic acid. The color intensity was colorimetrically measured at 405 nm with Beckman DU-64 UV/VIS spectrophotometer, USA (Moron et al., 1979).

Lipid peroxidation depended on the thiobarbituric acid reacting substance which is displayed as equivalents of malondialdehyde (MDA) using 1, 1, 3, 3-tetra methoxy propane as a standard (Buege and Aust, 1978). Quantification of NOx relied on the processing of nitrate to nitrite with copperized cadmium granules by a spectrophotometer at 540 nm (Ridnour et al., 2000).

### ELISA Assay in Hippocampal Tissue

The hippocampal tissue levels of TLR4, TNF-α, vascular endothelial growth factor-A (VEGF-A), and BDNF were evaluated by ELISA kits (Elabscience Biotechnology Inc., USA, Catalog No: E-EL-R0990, E-EL-R0019, E-EL-R2603, and E-EL-R1235, respectively). NF-kβ and Myd88 levels were also evaluated by ELISA kits (ELISA Genie Co., Dublin, Ireland, Catalog No: SKU: RTFI00988 and SKU: RTFI01303, respectively) based on the manufacturer’s instructions.

### Behavioral Test: The Forced Swimming Test

The FST was done based on earlier studies (Porsolt et al., 1978; Réus et al., 2011). The test was performed 24 h after the last treatment by placing the rat in a cylinder (60 cm in height × 25 cm in diameter) with 30 cm of water at 24°C–25°C, where the rodents cannot reach the bottom of the cylinder or exit. The overall duration of immobility was observed at a 6-min period. The rat was considered immobile when it remained floating in the water in an upright position with only very small movements to preserve its head above the water. The climbing time was calculated while the rat was doing an active movement on the side of the chamber with its forepaws in and out of water. The swimming time was calculated while the rat was doing a circular movement or active swimming. The decrease of the immobility duration and the increase of the swimming and climbing time indicate an antidepressant-like effect.

### Histological Studies of Brain Tissue

The brain of each animal was picked, split into two hemispheres to expose the hippocampi, rapidly fixed in 10% formalin solution for 24 h, embedded in paraffin, and sectioned in 5 µm thickness for the staining with hematoxylin and eosin (H&E) and the immunohistochemical staining (Suvarna et al., 2019).

### Immunohistochemical Studies of Brain Tissue

Immunohistochemistry was done for the assessment of glial fibrillary acidic protein (GFAP). Sections were deparaffinized, rehydrated, and then pretreated with 0.01% hydrogen peroxide to prevent the endogenous peroxidase action, submerged in 0.01 M citrate buffer (ph. 6) for 10 min for the antigenic site exposure followed by antigen retrieval in EDTA buffer in the microwave for 20 min, and incubated in the primary antibody anti-GFAP (polyclonal rabbit antibody, Catalog Number (G4546, 1:200) from Sigma-Aldrich Company, Egypt for 1 h in room temperature. Then, the sections were incubated in the avidin–biotin complex for 1 h. The sections were washed and incubated in peroxidase substrate (DAB) solution for 10 min. Finally, the sections were counterstained with hematoxylin, dehydrated in absolute alcohol, cleared by xylol, and mounted. The immunoreaction was detected as a dark brown cytoplasmic staining.

### Photography

The H&E and immunohistochemical sections were photographed using a high-resolution color digital camera mounted on a BX51 microscope (Olympus, Japan) and connected to a computer programmed with LC micro-application software. It was performed in the light microscopic unit of Histology and Cell Biology Department, Faculty of Medicine, Minia University.

### Morphometric Examination

The degenerated cell count (shrunken darkly stained) was obtained manually from the H&E stained sections. The GFAP immunoreaction was assessed under the 40X objective and evaluated as area fraction by image analysis software ImageJ (Schneider et al., 2012). The area fraction was examined in a standard measuring frame per 10 photomicrographs in each group, using a magnification of ×400 by a light microscopy. Areas having positively immunostained tissues were used for the evaluation, no matter the intensity of the staining. These areas were masked by a red binary color that could be measured by the computer system as follows:

1. Software converted the image type to an 8-bit greyscale.
2. The image was then color threshold to select only the color of interest, which is the brown color of the positive immunoreaction.
3. The color was then masked by a red binary color to measure the area fraction, which is the percentage of the pixels in the brown color that had been highlighted in red.

### Statistical Analysis

Results were expressed as the mean ± S.E.M. One-way analysis of variance (ANOVA) was performed and followed by the Tukey’s test to analyze the data for the statistically significant variance. The results of the forced swimming test were analyzed using the two-way ANOVA and the Tukey post hoc test. Significance was set at *p* values less than 0.05. The normal distribution of the quantitative variables was tested first by the Shapiro–Wilk test (*p* more than 0.05). GraphPad Prism software was used for statistical calculations (version 5.01 for Windows, GraphPad Software, San Diego California USA (www.graphpad.com).

## Results

### Influence of RVX at Different Doses on Monoamine Neurotransmitters

A significant reduction in 5HT, NE, and DA levels in the CUMS group was present in comparison with the control group. At the same time, all groups treated with either FLX or RVX at different doses displayed a significant elevation in their levels as compared to the CUMS group. RVXL still showed a significant decrease in monoamine neurotransmitters in comparison with the control and RVXH groups (Table 1).

**TABLE 1:** Effect of RVX at different doses on hypothalamic neurotransmitters levels.

	NEP (ng/mg protein)	Dopamine (ng/mg protein)	5-HT (ng/mg protein)
Control	40.3 ± 2.3	36.6 ± 1.9	18.6 ± 0.9
RVXL	38.3 ± 2.2	36.6 ± 2.5	17.6 ± 1.1
RVXH	37.6 ± 2.6	37.5 ± 3.2	18.2 ± 1.1
FLX/CUMS	37.8 ± 1.0	34.3 ± 2.0	17.4 ± 0.9
CUMS	16.4 ± 0.4^a^ ^,^ ^b^	15.9 ± 2.6^a^ ^,^ ^b^	7.2 ± 0.4^a^ ^,^ ^b^
RVXL/CUMS	30.9 ± 1.6^a^ ^,^ ^c^ ^,^ ^d^	27.1 ± 0.7^a^ ^,^ ^c^ ^,^ ^d^	14.3 ± 0.5^a^ ^,^ ^c^ ^,^ ^d^
RVXH/CUMS	39.5 ± 2.5^c^	37.6 ± 0.7^c^	18.4 ± 0.8^c^
*p* value	<0.0001	<0.0001	<0.0001

Findings are presented as mean ± S.E.M. Significant difference at *p* < 0.05.

a Significant difference compared to the control.

b Significant variance compared to the FLX group.

c Significant difference compared to the CUMS group.

d Significant difference compared to the RVXH.

CUMS, chronic unpredictable mild stress; DA, dopamine; FLX, Fluoxetine; 5-HT, serotonin; NE, norepinephrine; RVX L, rivaroxaban low dose; and RVXH, rivaroxaban high dose.

### Influence of RVX at Different Doses on Hippocampal Oxidative Stress Parameters

Compared to the control group, depression resulted in a significant reduction of the hippocampal GSH level and SOD activity, and increased the MDA and NOx levels. The administration of FLX or RVX in different doses significantly improved the oxidative stress parameters in comparison with the CUMS group. RVXL still showed a significant reduction of the hippocampal GSH level and SOD activity with elevated MDA and NOx levels when compared to the control and RVXH groups (Table 2).

**TABLE 2:** Influence of RVX at different doses on oxidative stress indicators in hippocampal tissue.

	SOD (U/g tissue)	MDA (nmol/g tissue)	GSH (μmol/g tissue)	NOx (nmol/g tissue)
Control	2,607 ± 173.9	17.6 ± 2.1	2,770 ± 49.1	26.8 ± 5.2
RVXL	2,482 ± 183.8	18.8 ± 2.3	2,707 ± 74.2	28.9 ± 5.2
RVXH	2,445 ± 178.7	18.8 ± 1.8	2,782 ± 31.8	27.4 ± 5.1
FLX/CUMS	2,228 ± 121.7	25.5 ± 2.9	2,636 ± 55.1	40.8 ± 3.6
CUMS	907.3 ± 25.5^a^ ^,^ ^b^	51.7 ± 2.2^a^ ^,^ ^b^	856.2.4 ± 120^a^ ^,^ ^b^	109.1 ± 6.0^a^ ^,^ ^b^
RVXL/CUMS	1,621 ± 106.9^a–d^	39.3 ± 1.8^a–d^	2,177 ± 86.2^a–d^	59.4 ± 5.1^a,c,d^
RVXH/CUMS	2,180 ± 84.7^c^	24.6 ± 2.4^c^	2,596 ± 97.2^c^	36.8 ± 3.9^c^
*p* value	<0.0001	<0.0001	<0.0001	<0.0001

Findings are presented as mean ± S.E.M. Significant difference at *p* < 0.05.

a Significant difference compared to the control.

b Significant variance compared to the FLX group.

c Significant difference compared to the CUMS group.

d Significant difference compared to the RVXH.

CUMS, chronic unpredictable mild stress; FLX, fluoxetine; GSH, reduced glutathione; MDA, malondialdhyde; NOx, total nitrite/nitrate; RVX L, rivaroxaban low dose ; and SOD, super oxide dismutase.

### Effect of RVX at Different Doses on Hippocampal NF-Kβ, Myd88, TLR4, TNF-α, VEGF-A, and BDNF Expression

**FIGURE 2:**
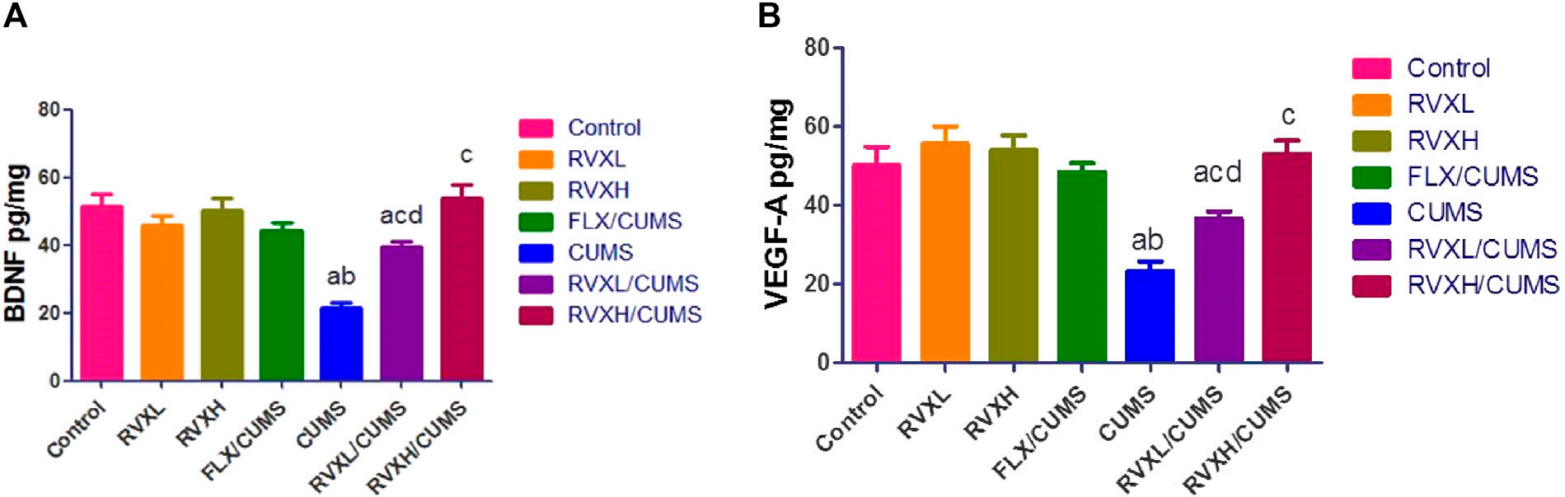
Effect of RVX at different doses on BNDF (Figure 1A) and VEGF-A (Figure 1B) in the hippocampal tissue. The findings are displayed as mean ± S.E.M. Significant difference at *p* < 0.05. ^a^significant difference compared to the control, ^b^significant difference compared to the FLX group, ^c^significant difference compared to the CUMS group, and ^d^significant difference compared to RVXH (BNDF: *p* < 0.0001, and *p* < VEGF-A: 0.0004). BNDF, brain-derived neurotrophic factor; CUMS, chronic unpredictable mild stress; FLX, fluoxetine; RVX L, rivaroxaban low dose; RVX H, rivaroxaban high dose; and VEGF-A, vascular endothelial growth factor-A.

**FIGURE 3:**
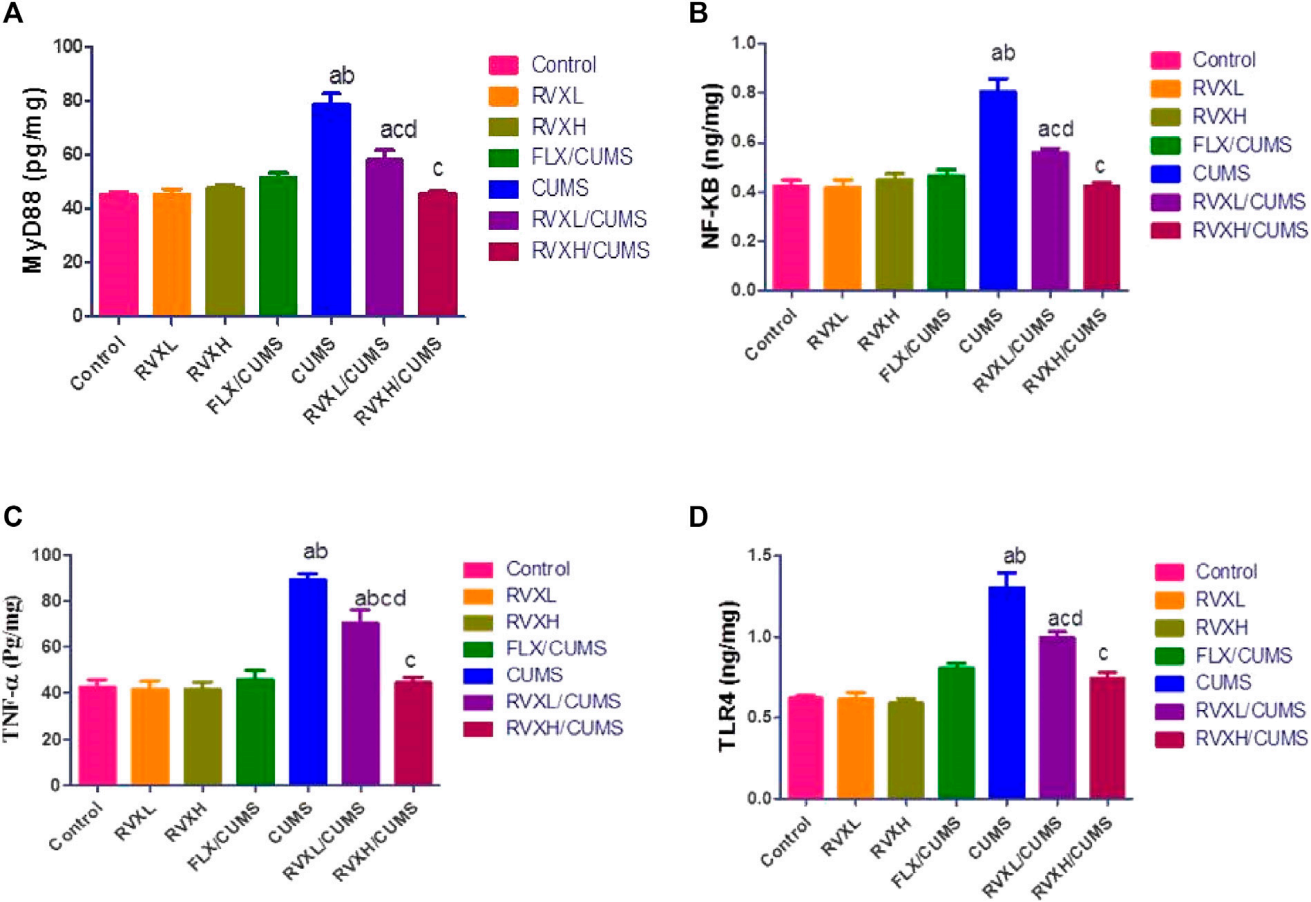
The findings are displayed as mean ± S.E.M. Significant difference at *p* < 0.05. ^a^significant difference compared to the control, ^b^significant difference compared to the FLX group, ^c^significant difference compared to the CUMS group, and ^d^significant difference compared to the RVXH (*p* < 0.0001). CUMS, chronic unpredictable mild stress; FLX, fluoxetine; NFkB, nuclear factor Kappa B; MyD88, myeloid differentiation factor 88; RVX L, rivaroxaban low dose; RVX H, rivaroxaban high dose; TLR4, Toll-like receptor 4; and TNF-α, tumor necrosis factor-α.

There was a significant decrease in the hippocampal VEGF-A and BDNF (Figure 2) with a significant increase in the hippocampal NF-kβ, Myd88, TLR4, and TNF-α (Figure 3). levels in the CUMS group when compared to the control group. 

The pretreatment with FLX or RVX at different doses showed a significant decrease in the hippocampal NF-kβ, Myd88, TLR4, and TNF-α and a significant increase in the hippocampal VEGFA and BDNF levels in comparison with the CUMS group. Meanwhile, RVXL still showed significantly increased hippocampal NF-kβ, Myd88, TLR4, and TNF-α and decreased hippocampal VEGF-A and BDNF in comparison to the control and RVXH groups (Figure 2, 3).

### Effects of RVX at Different Doses on Rats’ Behavior (FST)

**FIGURE 4:**
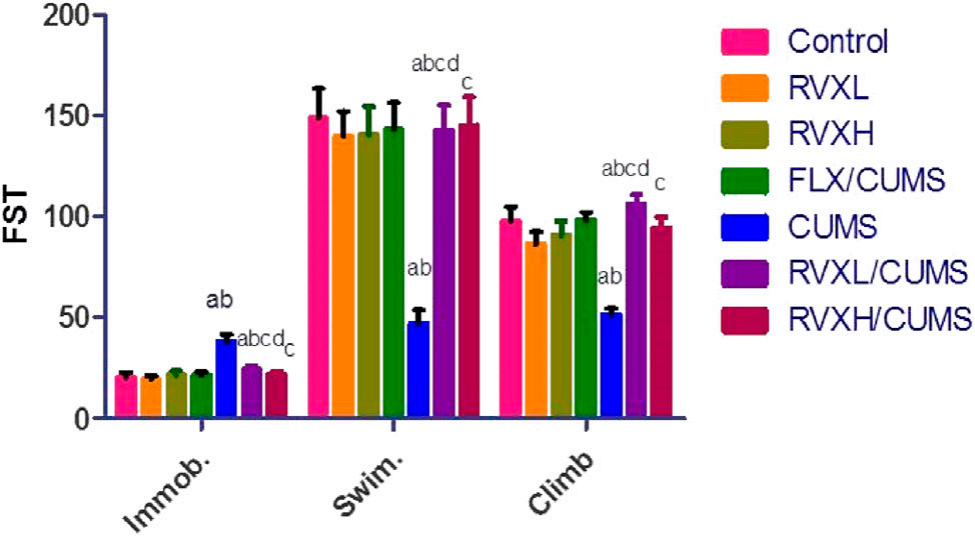
Effect of RVX at different doses on behavior (FST). The findings are displayed as mean ± S.E.M. Significant difference at *p* < 0.05. ^a^Significant difference compared to the control, ^b^significant difference compared to the FLX group, ^c^significant difference compared to the CUMS group, and ^d^significant difference compared to RVXH. *p* value <0.0001. CUMS, chronic unpredictable mild stress; FLX, fluoxetine; RVX L, rivaroxaban low dose; and RVX H, rivaroxaban high dose.

In Figure 4, depression caused a significant elevation in the immobility time with a significant reduction in the swimming and climbing times in comparison with the control group. Meanwhile, FLX and RVX at different doses revealed a significant reduction in the immobility time with significantly increased swimming and climbing times as compared with the CUMS group. RVXL still showed significant changes in behavior (FST) when compared with the control and RVXH groups.

### Effect of RVX at Different Doses on Hippocampal Histological Features

**FIGURE 5:**
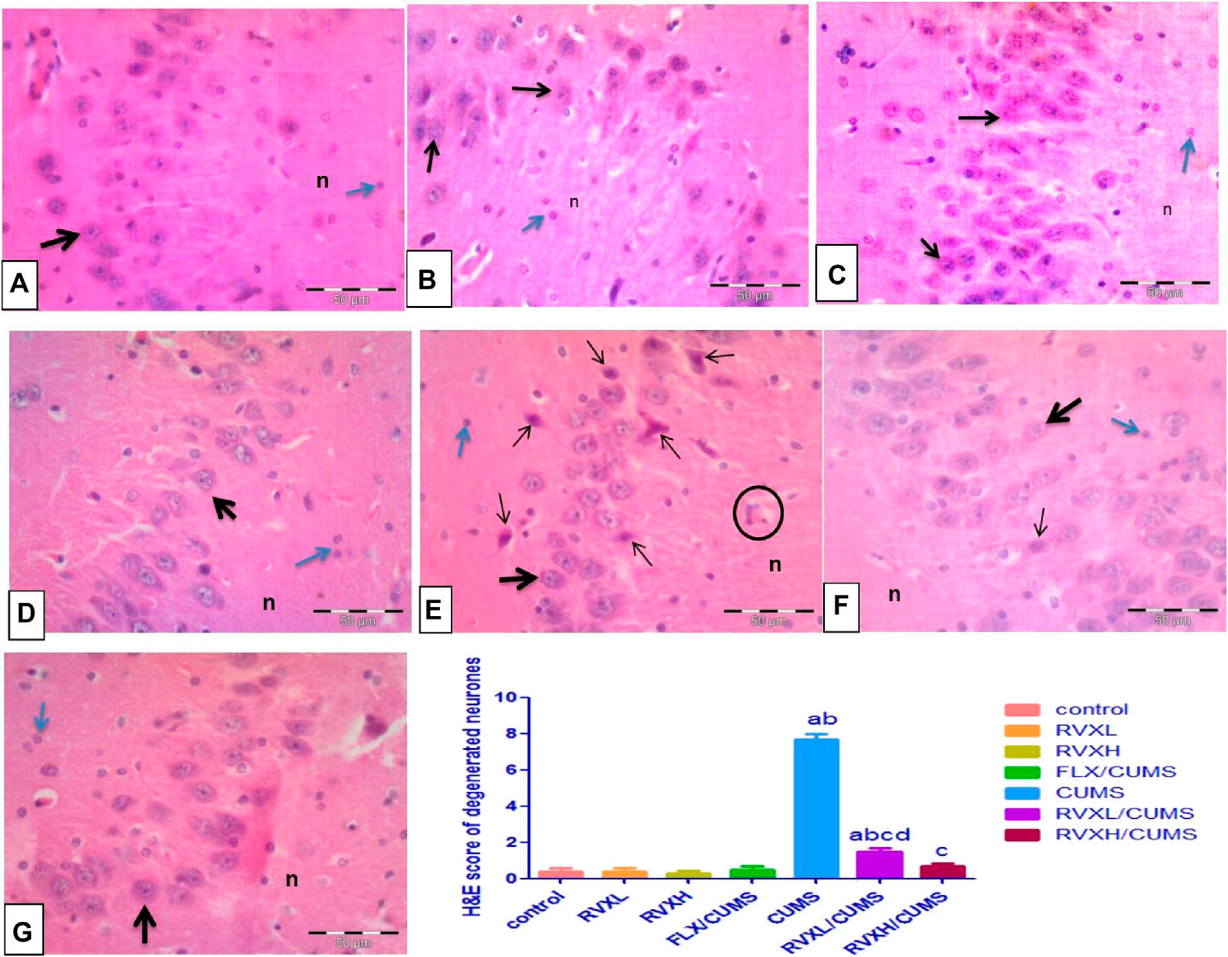
Representative photomicrographs of the CA3 zone of the hippocampus **(A––D)** Control, RVXL, RVXH, and FLX groups showing normal densely packed pyramidal neurons with vesicular nuclei and prominent nucleoli (thick arrow), neurophil (n), and glial cells (blue arrow). **(E)** The CUMS group showing shrunken elongated pyramidal neurons with darkly stained nuclei (thin arrows). The structure in the circle may indicate fused microglia. **(F)** The RVXL/CUMS group showing a normal morphological appearance of the pyramidal neurons (thick arrow), while the thin arrow reveals a shrunken elongated pyramidal neuron with a darkly stained nucleus. **(G)** RVXH/CUMS group showing normal neurons (thick arrow), neurophil (n), and glial cells (blue arrow). H&E x400, scale bars = 50 μm ^a^Significant difference compared to the control, ^b^significant difference compared to the FLX group, ^c^ significant difference compared to the CUMS group and ^d^significant difference compared to the RVXH. *p* value <0.0001. CUMS, chronic unpredictable mild stress; FLX, Fluoxetine; RVX L, rivaroxaban low dose; and RVX H, rivaroxaban high.

Cornu Ammonis 3 (CA3) cells seemed loosely packed with large pyramidal neurons with vesicular nuclei. Light eosinophilic neurophil background was observed, which contained neuronal and glial cell processes and sparse neuroglial cells in the control, RVXL, RVXH, and FLX groups (Figures 5A–D). Meanwhile, the hippocampus of the CUMS group showed distinct histological changes affecting the CA3 cells. There were multiple shrunken elongated pyramidal neurons with darkly stained nuclei and fused microglia (Figure 5E). The RVXL/CUMS–treated group showed ameliorated histopathological changes relative to the CUMS group, but there were few shrunken elongated pyramidal neurons with darkly stained nuclei (Figure 5F). Moreover, the normal cellular morphology was restored in the RVXH/CUMS–treated group (Figure 5G).

### Effect of RVX at Different Doses on Hippocampal GFAP Immunoreactivity (Astrogliosis)

**FIGURE 6:**
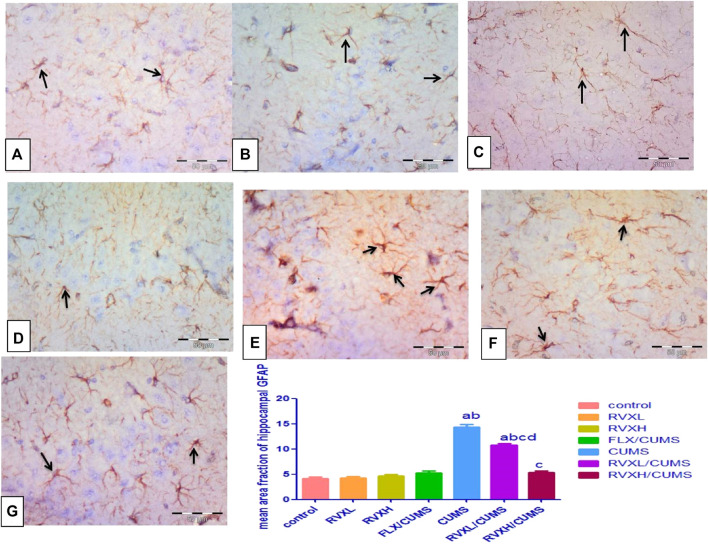
GFAP immunoreactivity. **(A––D)** Control, RVXL, RVXH, and FLX groups showing small-sized faintly stained astrocytes with few processes (arrows). **(E)** The CUMS group, displaying enlarged densely stained astrocytes with ramifying processes (arrows). **(F)** The RVXL/CUMS group, showing less densely stained astrocytes with fewer processes (arrows). **(G)** The RVXH/CUMS group, displaying more or less normal astrocytes (arrows). GFAP immunostaining x400, scale bars = 50 μm ^a^Significant difference compared to the control, ^b^significant difference compared to the FLX group, ^c^ significant difference compared to the CUMS group, and ^d^ significant difference compared to the RVXH. *p* value <0.0001. CUMS, chronic unpredictable mild stress; FLX, fluoxetine; RVX L, rivaroxaban low dose; and RVXH, rivaroxaban high.

The GFAP immunostaining revealed the astrocytes as star-shaped cells with thin processes. The control, RVXL, RVXH, and FLX groups displayed mild positive GFAP reactions in the astrocytes and their processes (Figures 6A–D). In the CUMS group, a strong GFAP immunoreaction was observed as enlarged densely stained astrocytes with prominent ramified cytoplasmic processes in comparison with the control and FLX groups (Figure 6E). In the RVXL-treated group, less-dense fewer GFAP astrocytes were seen (Figure 6F). Moreover, more or less normal astrocytes were noticed in the RVXH-treated group (Figure 6G).

## Discussion

Depression is a popular mental illness with a high incidence rate in the world. The pathophysiological mechanisms of depression are not refined, but many studies suggested that the impairment of intracerebral neurotransmission of 5-HT, NE, and DA is mainly accountable for the induction of depression (Du et al., 2020).

Depression reduces the 5-HT neural action and the 5-HT1A autoreceptor sensitivity. It leads to the alteration of 5-HT, NE, and DA receptors in number and sensitivity (Huang et al., 2019). Anhedonia in marked depression and hippocampal dysfunction in schizophrenia were related to the dopaminergic activity present in the nigrostriatal system, mesolimbic system, mesocortical system, and tuberoinfundibular system (Patton et al., 2013). The present study revealed a significant reduction of the monoamine neurotransmitters in the CUMS group, and this finding was previously proposed by Huang and his colleagues (2019).

Brain oxidative stress disturbance is a plausible pathogenesis and risk factor for behavioral disturbances and disorders (Moylan et al., 2013). Reactive oxygen species (ROS) lead to neuronal dysfunction through the oxidative injury to lipids, proteins, and nucleic acids (Ng et al., 2008).

The current results showed a marked oxidative disequilibrium. It is evident by the significant increase in MDA and NOx and the reduction in SOD and GSH in the CUMS group. Xu et al. (2014) confirmed that increased oxidative stress had co-existed with depression. CUMS was reported to damage the oxidative equilibrium of the brain tissue through the elevated ROS production that strongly affected the limbic system. SOD is the main antioxidant enzyme essential for superoxide detoxification. It catalyzes the dismutation of the superoxide radical to hydrogen peroxide and oxygen. Superoxide, a byproduct of oxygen metabolism, can cause many types of cell damage and SOD, known to be a part of antioxidant defenses against superoxides, and has been demonstrated to reduce plasma MDA levels (Wang et al., 2015). GSH is another endogenous antioxidant that plays a role in the elimination of hydrogen peroxide generated by SOD (Glantzounis et al., 2005). The suppressed expression of SOD in the hippocampus was observed in stressed animals (Xu et al., 2014). Zhang et al., 2009 and his colleagues in 2009 reported that CUMS decreased the total antioxidant capacity (TAC), GSH content, and SOD activity.

RVX antioxidant activity was explained by scavenging the oxidative products generated by depression effect and this is concordant with Al-Harbi et al., 2020, who proved that RVX significantly increased GSH with a decrease in the MDA intracellular levels in sunitinib-induced renal injuries in rats. RVX exerted its effect via the increased utilization of antioxidants. The reduction of the oxidant MDA in tissues might be related to a direct action of RVX (Özbudak et al., 2019). This was noticed in the present research, where the co-administrated RVX ameliorated the oxidative stress indicators.

Oxidative stress stimulates numerous intracellular signaling pathways causing the upregulation of proinflammatory cytokine production (Aladaileh et al., 2019). A pivotal mechanistic link between oxidative stress and inflammation elevates the proinflammatory cytokines, for example, NF-kβ and TNF-α (Rao et al., 2011). Also, the inflammatory cytokines and cytokine producers influenced the metabolism of 5-HT, DA, and NE (Anisman et al., 2008).

The current experiment showed that CUMS significantly increased the hippocampal NF-kβ, Myd88, TLR4, and TNF-α levels. Given that TLR is a clef regulator of inflammation, the activation of TLR4 via MyD88 and the subsequent stimulation of NF-κB translocation into the nucleus up-regulated several proinflammatory factor levels, for example, IL-1β and TNF-α. It is worth mentioning that TLR4 regulates inflammation and tissue injury in different animal models, for example, ischemia/reperfusion, Alzheimer’s disease, and autoimmune disorders (Liu et al., 2020). Lai et al. (2018) reported that TLR4 had increased in the basolateral amygdala of posttraumatic stress disorder in rats.

The co-administration of RVX decreased the inflammatory parameters significantly when compared to the CUMS group. RVX could protect the experimental animals from inflammation by blocking the factor Xa which is considered as an accelerator for the generation of proinflammatory mediators. Many researchers have confirmed the potential anti-inflammatory role of RVX. The previous studies of Jhaveri et al. (2011), Basile et al., 2012 and his colleagues in 2012, Ma et al., 2018 and his coworkers in 2017, and Al-Harbi et al. (2020) were in line with the above-discussed literature that RVX has anti-inflammatory effects. Also, RVX dampened the inflammatory response in the ischemic brain (Dittmeier et al., 2016).

RVX suppressed the transcription of NF-κB by inhibiting TLR4 expression that negatively regulated the Myd88-dependent TLR4/NF-κB signal transduction decreasing the inflammatory response. RVX reduced the NF-κB activity through the Myd88-dependent pathway of TLR4.

CUMS mediated the oxidative damage–induced activation of NF-κβ which results in DNA fragmentation and ultimately increased neuronal death through apoptosis or other mechanisms that are responsible for the observable behavioral deficits (Tiwari and Chopra, 2012; Tiwari et al., 2012). Also, NF-κB mediated inflammatory signaling in the brain of rats chronically administered with ethanol (Tiwari and Chopra, 2013).

Kuhad et al., 2009 and his colleagues reported that the NF-κB signaling pathway is associated with diabetes-induced cognitive impairment and points towards the therapeutic potential of tocotrienol in diabetic encephalopathy.

Lou et al. (2019) supported our finding as they stated that RVX had anti-atherosclerotic effects by regulating the expression of the genes in the TLR4/NF-κB signaling pathway and the activation of the downstream molecules.

BDNF and VEGF are neurotrophins that showed a stress-induced drop when the neurogenic/neurotrophic hypothesis of depression is implicated. The presented results showed a significant decrease in BDNF and VEGF levels in the CUMS group. The VEGF function was closely related to the NE system as it affected the monoamine levels (Udo et al., 2014). The decrease in the VEGF level detected in the present study is in line with the findings obtained by Howell et al. (2011) and Nowacka-Chmielewska et al., 2017 and his colleagues in 2017. BDNF has a function in neuronal differentiation, survival, maintenance, and synaptic plasticity. Its deficiency is shared in the pathogenesis of depression. It has been reported that BDNF activates tyrosine kinase B (Trk B) increasing the 5HT level. Also, the 5HT expression increases after the increase in the BDNF expression (Sun et al., 2020).

RVX increased the BDNF and VEGF levels significantly in the present study opposing the effect of CUMS. RVX directly affected the endothelial progenitor cells (EPCs) to release VEGF, thereby promoting neovascularization in diabetic mice (Wu et al., 2015). The current findings are in harmony with those of Hollborn et al. (2012), who reported that the factor Xa increased the chemotaxis of retinal pigment epithelial cells with the stimulation of VEGF. RVX, the factor Xa inhibitor, suppressed the coagulation-induced angiogenesis by directly affecting the release of VEGF from the EPCs. The BDNF and 5HT can promote each other and RVX can upregulate the BDNF level in the depressive rats by regulating the serotonin system. The FST was performed and our results were confirmed. It is one of the most commonly used assays for the study of depression (Réus et al., 2011). The probable antidepressant activity of RVX was evaluated by the FST.

The histopathological studies confirmed our biochemical results. The microglial activity was linked to pathological conditions like stress, pathological aging, and chronic infections. These cells are primary immune effector cells in the CNS that regulate the interaction between the nervous and the immune systems as a reaction to various stressors. Research studies proposed that during stress, the microglia have a paramount effect in the impairment of neuroplasticity and injuriously affect the neuroprotection resulting in neuroinflammation and induction of depression. Although many theories have been supposed for the microglial effect in producing depression, it is obvious that all molecular pathways to depression are related to the microglia-associated neuroinflammation and hippocampal dysfunction (Baune and Singhal, 2017).

Besides the adjustment of immune activity, the microglia adjusts some neurobiological functions as the synthesis of neural circuits (Wake et al., 2013) and synapses (Kettenmann et al., 2013) in infants, and phagocytose apoptotic cells in adults (Sierra et al., 2010). Moreover, the microglia regulated the levels of neurotrophic (Nakajima et al., 2007), angiogenesis factors (Rymo et al., 2011), and amino acid metabolism (Gras et al., 2012) in the brain. All these activities are pivotal for the neuroplasticity and they are negatively affected by the suppressed number or functions of the microglia. Another cause affecting the microglial shape is the excessive glucocorticoids, and it also caused depression (Nair and Bonneau, 2006; Marques et al., 2009). RVX revealed an improvement in the histopathological changes observed in depression.

Appropriate astrocyte–microglia cross-talk in disease is essential for the astrocytes to support neuronal survival and function after acute injury (Hanisch and Kettenmann, 2007). Secondary to microglial reaction is the activation of astrocytes, which release inflammatory mediators that signal to the microglia and can recruit infiltrating blood cells including monocyte-derived macrophages. Reactive astrocytes upregulate GFAP and undergo morphological changes leading to the construction of glial scars, which may limit damage within the affected area (Sofroniew, 2014).

The astrocyte is obviously activated in prefrontal cortex and hippocampus in the inflammation-induced depression by lipopolysacchride (LPS). Inhibition of activated astrocytes improves LPS-induced depressive-like behavior, providing the first confirmation that the inhibition of activated astrocytes might denote a novel therapeutic target for depression (Wang et al., 2018).

The current study showed that RVXH is more protective against depression than RVXL as RVXL is still significant from control despite its significance from CUMS.

## Conclusion

RVX, in a dose-dependent manner, possesses potent ameliorative effects against depression by decreasing oxidative stress, inflammation, and regulating the TLR4/Myd88/NF-kβ signaling pathway. These results can shed light on the clinical implication of its use to treat depression.

## Data Availability

The raw data supporting the conclusion of this article will be made available by the authors, without undue reservation.

## Abbreviations

BDNF, brain-derived neurotrophic factor; CUMS, chronic unpredicted mild stress; DA, dopamine; FST, forced swimming test; GFAP, glial fibrillary acidic protein; GSH, reduced glutathione; 5-HT, serotonin; IL, interleukin; MDA, malondialdehyde; MyD88, myeloid differentiation factor 88; NE, norepinephrine; NF-κB, nuclear factor-kappa B; NOx, total nitrite/nitrate; p.o., per oral; ROS: Reactive oxygen species; RVX, rivaroxaban; SOD, superoxide dismutase; TLR4, Toll-like receptor 4; TLRs, Toll-like receptors; TNF-α, tumor necrosis factor-α; and VEGF-A, vascular endothelial growth factor-A.
